# Parapharyngeal Schwannoma: Limitations Regarding Diagnosis and Surgical Intervention—A Case Report and Focused Literature Review

**DOI:** 10.3390/diagnostics16182906

**Published:** 2026-09-09

**Authors:** Despina Luciana Bereczki-Temistocle, Cecilia Petrovan, Adina Simona Coșarcă, Mihai Vlad Golu, Gabriela Felicia Bereșescu, Bianca-Gabriela Nenec, Andrei Cosmin Nenec, Alina Ormenișan

**Affiliations:** 1 Department of Oral and Maxillofacial Surgery, George Emil Palade University of Medicine, Pharmacy, Science and Technology of Târgu Mureș, 540139 Târgu Mureș, Romania; despina.bereczki-temistocle@umfst.ro (D.L.B.-T.); cecilia.petrovan@umfst.ro (C.P.); adina.cosarca@umfst.ro (A.S.C.); vlad.golu@umfst.ro (M.V.G.); alina.ormenisan@umfst.ro (A.O.); 2 Department of Tooth and Dental Arch Morphology, George Emil Palade University of Medicine, Pharmacy, Science and Technology of Târgu Mureș, 540139 Târgu Mureș, Romania; felicia.beresescu@umfst.ro; 3 Department of Otorhinolaryngology, M3—Department of Clinical and Medico-Surgical Sciences, George Emil Palade University of Medicine, Pharmacy, Science and Technology of Târgu Mureș, 540142 Târgu Mureș, Romania; vaduva.bianca-gabriela.25@stud.umfst.ro; 4 Department of Stomatology and Maxillofacial Surgery, Târgu Mureș Emergency County Hospital, 540136 Târgu Mureș, Romania

**Keywords:** parapharyngeal space, schwannoma, neurilemmoma, nerve sheath tumor, vagus nerve, cervical sympathetic chain, case report, literature review

## Abstract

**Background:** Parapharyngealspace schwannomas are rare benign tumors that may become significantly enlarged prior to producing symptoms. Their deep anatomical location and proximity to major neurovascular structures may pose significant challenges for diagnosis and surgical management. **Case Presentation:** A 51-year-old woman presented with a slowly enlarging, painless right-side neck mass. Contrast-enhanced CT-scan demonstrated a large, well-circumscribed post-styloid parapharyngeal mass measuring approximately 44 × 53 × 73 mm, displacing rather than invading the adjacent carotid vessels. Paraganglioma was included in the differential diagnosis. MRI-scan was not performed because the CT-scan provided sufficient anatomical information for surgical planning in our clinical setting. The tumor was completely removed through an extended transcervical–cervico-parotidial approach without capsular lesions. The origin nerve was not identified intraoperatively. The histopathological exam confirmed a benign schwannoma. No clinically apparent neurological deficit was observed at the 6- and 12-months follow-ups. **Literature Review:** A focused literature review of PubMed, Scopus, and Web of Science was performed to contextualize the clinical findings and surgical management. The literature highlights the difficulty of determining the nerve of origin preoperatively and the overlap between post-styloid schwannomas and paragangliomas on imaging studies. Surgical excision remains the main treatment, although evidence regarding optimal surgical technique, long-term functionality and disease-control outcomes remain controversial. **Conclusions:** This case-study highlights the diagnostic challenges of large post-styloid parapharyngeal schwannomas and the limitations of imaging studies in establishing the nerve of origin. Surgical management should be individualized according to tumor anatomy, spatial neurovascular relationships, and anticipated functional risks.

## 1. Introduction

The parapharyngeal space (PPS) is a deep, inverted-pyramidal virtual space of the suprahyoid portion of the neck extending from the skull base to the greater cornu of the hyoid bone. The tensor-vascular-styloid fascia divides it into a pre-styloid compartment, containing fat, the retromandibular portion of the deep parotid lobe and minor salivary tissue, and a post-styloid (retro-styloid) compartment, containing the internal carotid artery (ICA), the internal jugular vein (IJV), the IX–XII cranial nerves and the cervical sympathetic chain [1]. This compartmental anatomy governs both the differential diagnosis of PPS masses and the surgical strategy required to remove them safely. The main radiological differentiators of the pre- and post-styloid spaces are presented in Table 1 [2,3].

Tumors of the PPS are rare, accounting for approximately 0.5% of head and neck neoplasms [2,4]. About half of them are of salivary-gland origin—most commonly pleomorphic adenoma—approximately 20% of which are neurogenic, and the remainder comprise lymphoreticular lesions, paragangliomas, metastases and rarer entities. Among neurogenic tumors, the schwannoma (neurilemmoma) is the most frequent benign nerve-sheath neoplasm [4,5]. Schwannomas are slow-growing, encapsulated tumors originating from the Schwann cells of the neural sheath and may stem from any cranial, peripheral or autonomic nerve [6]; within the post-styloid PPS though, the vagus nerve and the cervical sympathetic chain predominate [7].

Because of their indolent growth, PPS schwannomas frequently remain asymptomatic until they reach a considerable size, when they present as a painless neck or oropharyngeal mass or produce pressure symptoms such as dysphagia, globus, muffled voice or, less commonly, cranial-nerve or sympathetic deficits [5,8]. Their deep location, proximity to the carotid sheath and lower cranial nerves, and non-specific imaging appearance make both preoperative diagnosis and surgical excision demanding. Fine-needle aspiration (FNA) cytology is often inconclusive, and the diagnosis is typically confirmed histopathologically [9].

Given the rarity of these lesions, the published evidence consists largely of single-institution case series and individual case reports [10,11], and management has evolved substantially—most notably from radical resection toward function-sparing intracapsular enucleation [12].

Despite advances in cross-sectional imaging, the preoperative diagnosis and surgical planning of parapharyngeal schwannomas remain challenging, particularly for large post-styloid lesions. Imaging studies may suggest the compartment of origin and demonstrate relationships with the carotid and jugular vessels, but it does not always reliably distinguish schwannoma from paraganglioma or establish the origin nerve. This uncertainty is clinically relevant because the nerve of origin may determine the risk of postoperative neurological dysfunction and influence the choice of surgical approach and extent of resection. The present case addresses this clinical gap by illustrating a large post-styloid parapharyngeal schwannoma that was radiologically considered compatible with paraganglioma, in which the parent nerve could not be identified preoperatively or intraoperatively, yet complete excision was achieved without clinically apparent postoperative neurological deficit. The accompanying focused literature review places this diagnostic and surgical challenge in the context of the available evidence and highlights the limitations of imaging-based prediction of nerve origin and the need for individualized surgical planning.

## 2. Detailed Case Description

This case is reported in line with the CARE (CAse REport) guidelines.

### 2.1. Patient Information and Presentation

A 51-year-old woman was electively admitted to the Department of Oral and Maxillofacial Surgery, presenting for a right submandibular and posteroinferior parotid-region swelling associated with spontaneous pain. Our initial evaluation was performed at a private outpatient polyclinic (SC ALGOCALM SRL, Târgu Mureș, Romania), where the parapharyngeal mass was clinically identified, and referral for imaging and surgical management was arranged. The referring diagnosis was an unspecified right parotid tumor of uncertain behavior.

The patient had first noticed a small right submandibular mass approximately 7–8 years prior. At that time the lesion produced no clinically-evident functional impairment. According to the patient’s account at admission and to the referral letter available to us, she was evaluated by an otorhinolaryngologist and the initial imaging assessment showed no findings considered to require immediate intervention; clinical and imaging surveillance was therefore recommended. Neither the original imaging studies nor the corresponding radiological reports were available for review, so these findings could not be independently verified. The mass presented continuous, slow enlargement documented by the patient over the years, and, in the 3–4 months prior to admission, became more prominent and painful, prompting renewed evaluation and surgical treatment. The available records (presented by the patient, from the initial assesment) documented no clinical signs of dysphagia, odynophagia, nasal obstruction, voice change, referred otalgia or other significant oropharyngeal symptoms, and the patient reported no sensory or other neurosensory disturbance.

The medical history indicated type A hepatitis during childhood and GOLD stage I–II chronic obstructive pulmonary disease (COPD). The patient was an active smoker of approximately one pack per day, denied alcohol consumption and reported no chronic treatment.

### 2.2. Clinical and Neurological Examination

At admission the patient presented an adequate general condition, with no apparent clinical deficits. General examination revealed no acute cardiovascular, gastrointestinal or genitourinary abnormalities, and the respiratory examination was consistent with the known mild-to-moderate COPD without acute auscultatory findings. Inspection showed facial asymmetry caused by a right submandibular and posteroinferior parotid-region swelling with right latero-cervical extension; the overlying skin and adjacent mucosa were normally colored, without ulceration, inflammatory change or signs of infiltration. On palpation the lesion appeared oval-shaped, well circumscribed, with clear margins and a firm-elastic consistency; it had preserved mobility relative to the superficial and deep planes and was nontender. Multiple prominent, non-tender right submandibular lymph nodes were noted. Intraoral examination revealed multiple dental infectious foci and residual roots. The lymph nodes appeared clinically reactive, most likely related to the intraoral dental infectious foci. They were assessed on the contrast-enhanced CT examination and showed morphological features consistent with benign reactive adenopathy. No dedicated imaging follow-up was performed; the nodes were monitored clinically by palpation and had regressed at reassessment one week later.

No clinical preoperative facial weakness or other neurosensory abnormality was identified. The records documented neither Horner syndrome nor deficits of cranial nerves IX–XII; no palatal dysfunction, tongue deviation, dysphonia or swallowing impairment was reported or observed. Because flexible laryngoscopy was not performed, vocal-folds mobility itself was not objectively assessed, and the absence of clinically apparent voice changes cannot be equated with normal vocal-fold function.

### 2.3. Imaging Evaluation

Preoperative clinical and paraclinical assessment included cardiological and pulmonary investigations, as well as full blood work to assess the patient’s general status. No clinically relevant preoperative impairment of swallowing or phonation was documented.

**Figure 1:**
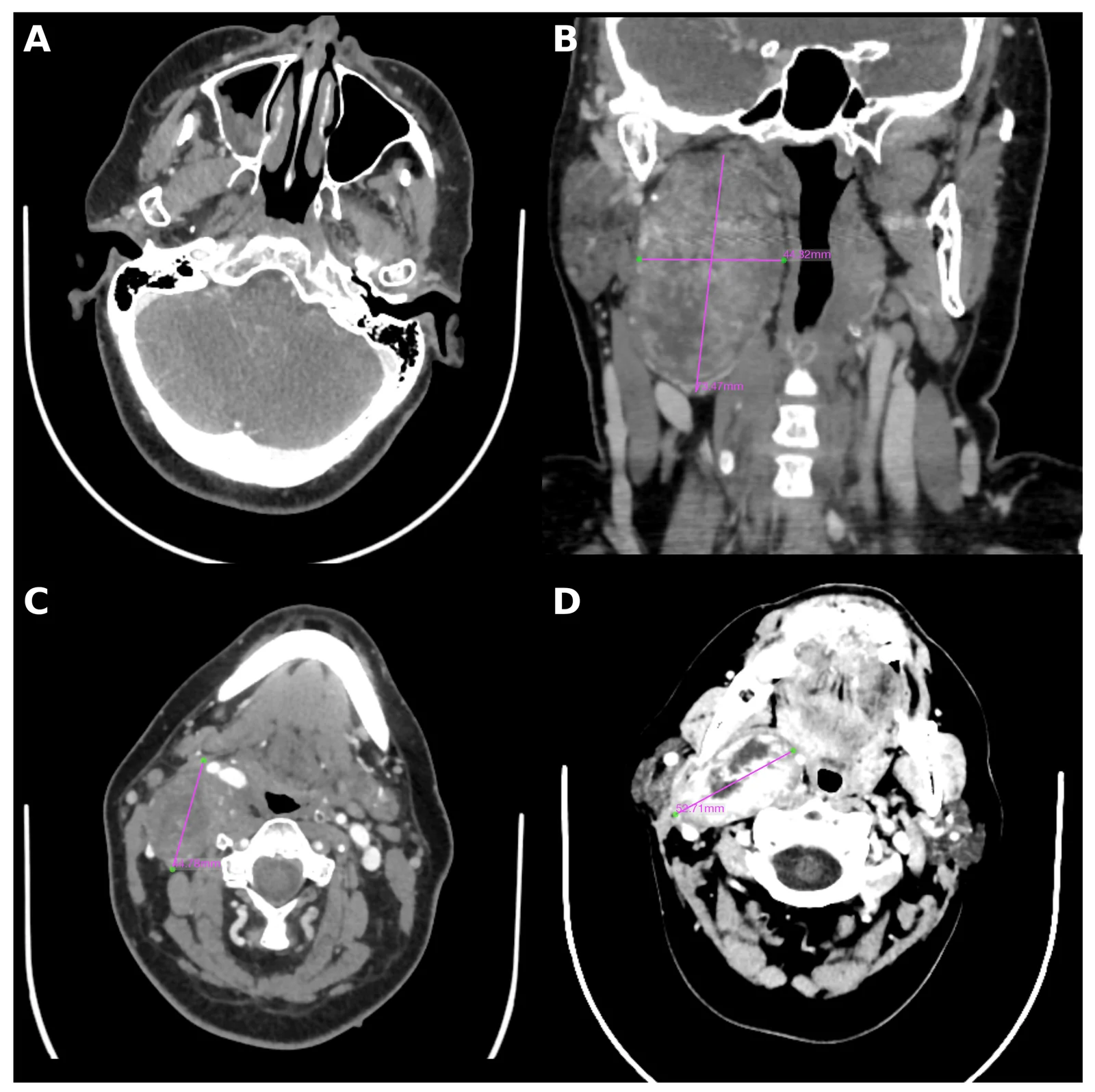
Contrast-enhanced CT scans of the neck were obtained using RadiAnt DICOM Viewer (version 2026.1; Medixant, Poznań, Poland). (**A**) Axial image at the skull-base level showing the superior component of the right parapharyngeal mass. (**B**) Coronal arterial-phase reconstruction showing the large craniocaudal extent of the well-defined lesion (approximately 44 mm maximal transverse and 73 mm craniocaudal diameter) and medial displacement of the pharyngeal lumen. (**C**,**D**) Axial contrast-enhanced images showing maximal transverse diameter of approximately 53 mm, heterogeneous enhancement and a close relationship with the right carotid–jugular structures. The images were anonymized for publication. The pink lines represent the measurement segments, while the green dots indicate the corresponding measurement endpoints.

Contrast-enhanced CT scan demonstrated a large, well-circumscribed, oval-to-lobulated right parapharyngeal soft-tissue mass measuring approximately 44 × 53 × 73 mm (Figure 1). Measurements were performed using RadiAnt DICOM Viewer (version 2026.1; Medixant, Poznań, Poland). The lesion was centered predominantly in the post-styloid compartment, with anterior extension into the pre-styloid space and cranio-caudal extension from the skull-base region to the submandibular and hyoid level. It showed heterogeneous contrast enhancement with small internal areas of lower attenuation, concordant to the cystic degenerative change subsequently identified histologically. Although a paraganglioma was included in the radiological differential diagnosis, the lesion did not demonstrate imaging features suggesting a highly vascular tumor that would have prompted additional vascular imaging in the clinical context. Based on these findings, and considering the apparently non-invasive and well-demarcated nature of the lesion, additional MRI scan was not considered necessary for the immediate diagnostic and surgical planning.

The mass produced marked local mass effect, with medial displacement of the right lateral pharyngeal wall and left deviation and narrowing of the pharyngeal airway. It extended laterally toward the parotid region without an unequivocal intra-parotid origin and was closely related to the right carotid–jugular structures, with postero-inferior displacement of the IJV, anterior displacement of the ICA and displacement of the external carotid artery toward the hyoid region. No circumferential vascular encasement, major luminal occlusion or adjacent osseous erosions were identified.

The combination of post-styloid location, heterogeneous enhancement and marked vascular displacement led to a preoperative radiological suspicion of paraganglioma. In retrospect, the well-defined margins, internal heterogeneity with degenerative areas and displacement rather than infiltrative encasement of adjacent structures were compatible with a nerve-sheath tumor. The exact nerve of origin could not be determined from the available images. Neither MRI nor FNA were performed for this patient, and no angiographic evaluation was undertaken. Flexible laryngoscopy was not performed preoperatively; therefore, baseline vocal-fold mobility was not objectively documented.

### 2.4. Surgical Treatment

Complete excision of the right parapharyngeal tumor was performed under general anesthesia with orotracheal intubation and continuous monitoring of vital functions. The tumor was approached through a modified Blair incision, corresponding to an extended cervico-parotid (transcervical) exposure placed around the right mandibular angle and extended towards the submandibular and latero-cervical regions.

**Figure 2:**
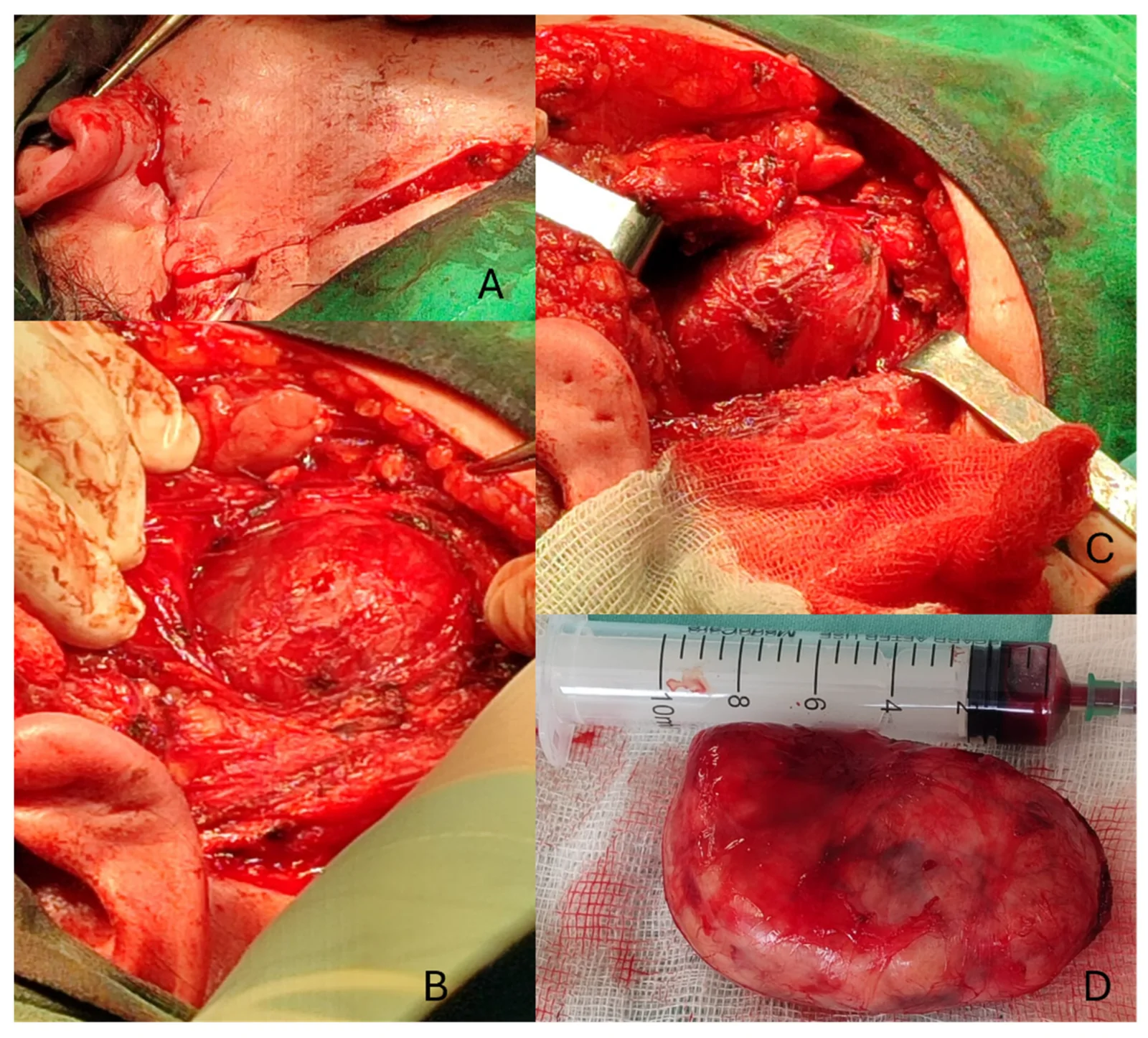
Intraoperative and gross findings. (**A**) Extended transcervical/cervicoparotid exposure of the right neck, with the tumor bed opened and a suction drain in place. (**B**) The well-encapsulated parapharyngeal tumor exposed in the poststyloid space and retracted. (**C**) Blunt and sharp dissection of the tumor capsule from the adjacent structures. (**D**) The excised specimen—a well-encapsulated, ovoid tumor with a heterogeneous, focally cystic and hemorrhagic surface—shown against a 10 mL syringe for scale. The patient’s facial features were cropped from panel (**A**) for anonymization; written informed consent for publication of the clinical images was obtained.

After dissection through the skin, platysma and cervical muscle planes, the vascular compartment was explored. An approximately 7 cm, well-encapsulated tumor was identified in the right post-styloid parapharyngeal space, with an extension into the pre-styloid compartment. The lesion was closely related to the right IJV, which was pushed posteroinferior to the tumor, the ICA, which was displaced anteromedially; and the ECA, which was displaced medial to the pharyngeal mucosal space and anteromedial to the prevertebral space. Laterally, it extended toward the superficial lobe of the right parotid gland, although no direct origin from or macroscopic continuity with the gland was identified (Figure 2). Pericapsular dissection was performed and the tumor was completely removed intact without capsular rupture. The origin nerve was not identified intraoperatively; the tumor, being large, encapsulated and occupying the post-styloid parapharyngeal space, with no clearly identifiable nerve entering or leaving the mass during the surgical exposure; unfortunately intraoperative nerve monitoring is not available in our center; based on the vascular displacement pattern, a vagal origin was considered likely. After hemostasis the wound was closed in anatomical layers, a drain was placed in the right retro-auricular and cervical region and a compressive dressing was applied.

### 2.5. Histopathological Findings

**Figure 3:**
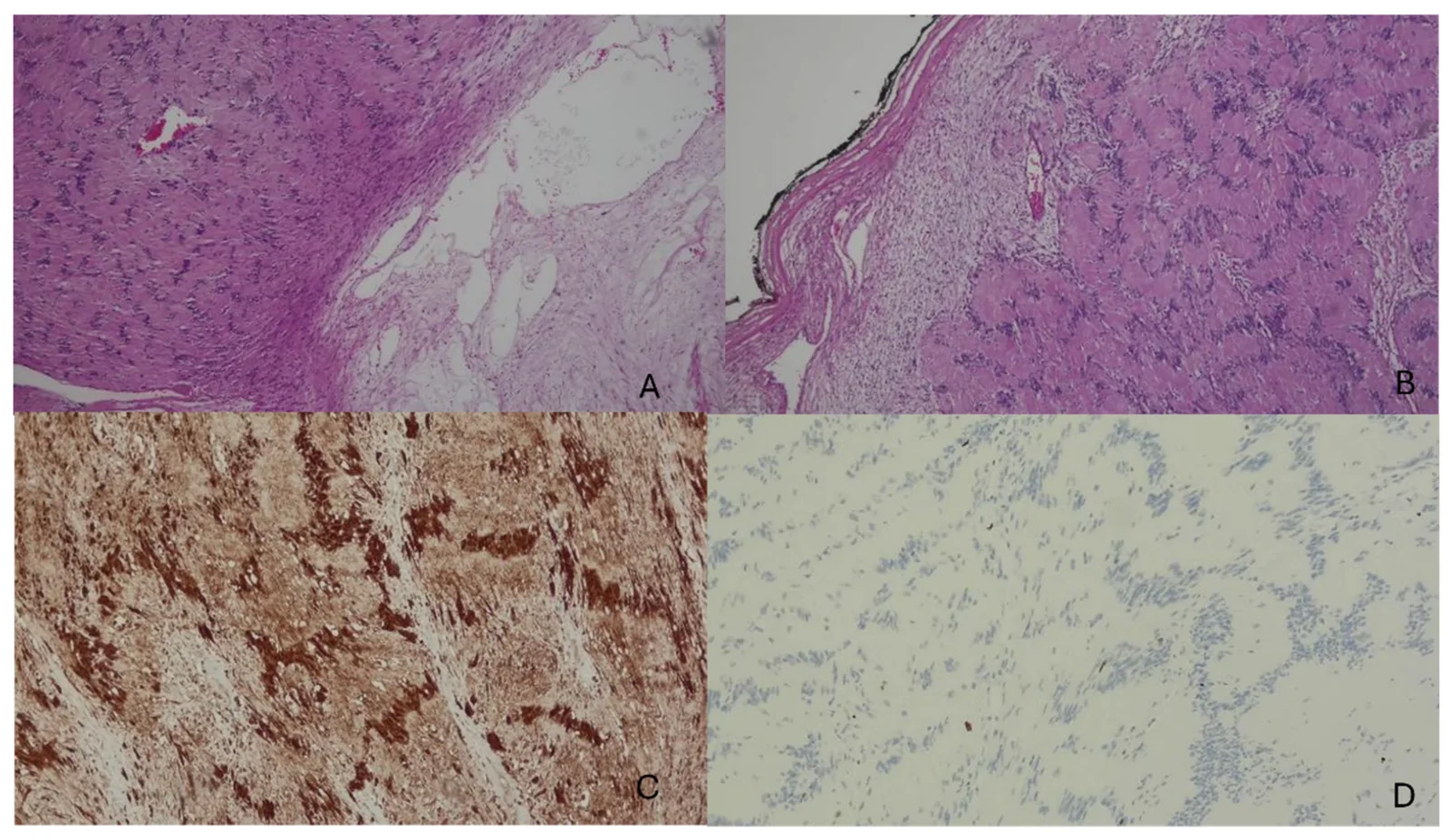
Histopathological findings. (**A**) H&E staining showing hypercellular Antoni A areas with Verocay bodies alternating with hypocellular Antoni B areas (×10). (**B**) H&E staining showing the peripheral capsule and an adjacent Antoni A area with Verocay bodies (×5). (**C**) Diffuse S-100 immunopositivity (×10). (**D**) Low Ki-67 proliferation index (×10).

The gross examination showed a nodular specimen measuring 65 × 45 × 30 mm of elastic consistency, with an intact external capsule. The radiological dimensions represent measurements obtained in vivo on cross-sectional imaging, whereas the pathological specimen measurements were obtained after excision and tissue contraction; therefore, direct comparison is not expected to be identical. The cut surface was heterogeneous and grey-white to yellow-white, with areas of cystic degeneration and punctate hemorrhagic foci. Microscopically, the tumor was well circumscribed and encapsulated and displayed the characteristic biphasic architecture: hypercellular Antoni A areas composed of elongated cells with eosinophilic cytoplasm and elongated, monomorphic nuclei, alternating with more extensive hypocellular Antoni B areas of loose architecture, cells with clearer cytoplasm and rounded nuclei, and cystic degenerative change. Focal nuclear palisading with Verocay body formation was present. Immunohistochemically, the tumor cells showed diffuse, strong positivity for S-100 protein and a low Ki-67 proliferation index (no numerical estimates were available); SOX10 staining was not performed. The final diagnosis was benign schwannoma (ICD-O 9560/0), and it was completely excised (Figure 3).

### 2.6. Postoperative Course and Follow-Up

The postoperative course was favorable. Drainage was minimal and serosanguineous, and no major perioperative complication occurred. The patient received clinical monitoring, intravenous analgesia, antimicrobial and supportive treatment, and local wound care. No clinically apparent postoperative cranial-nerve deficit was documented. The patient was discharged in improved general condition, with the surgical wound considered fully healed, with recommendations for local wound care, short-term anti-inflammatory treatment, avoidance of local trauma, suture removal and outpatient reassessment once the definitive histopathological result was available. The patient was reexamined clinically at 6 and 12 months postoperatively. At both visits the surgical site was fully healed, with no palpable residual or recurrent mass and no new clinically no new or persisting cranial-nerve deficit. Assessment at both visits comprised a structured clinical interview together with a targeted cranial-nerve examination performed by the treating team. Vagal and glossopharyngeal function was assessed indirectly by inspection of palatal elevation and by voice quality and water-swallow testing; hypoglossal function by tongue protrusion; accessory-nerve function by shoulder elevation and sternocleidomastoid strength; facial-nerve function by testing of the mimic musculature; and sympathetic-chain integrity by inspection for ptosis, miosis and facial anhidrosis. All these examinations were normal. First-bite syndrome and voice changes were ascertained by directed history alone, since they are symptom-defined, and the patient reported neither. Because flexible laryngoscopy was not performed, vocal-fold mobility was not objectively documented at any point. Neither revision surgery nor any adjuvant oncological treatment was required. At the most recent follow-up, 12 months postoperatively, the patient remained asymptomatic and free of clinically detectable disease, and she continues under periodic clinical surveillance. Follow-up was clinical only; no postoperative cross-sectional imaging or flexible laryngoscopy was performed, so a subclinical vocal-fold motion deficit cannot formally be excluded.

## 3. Literature Review

A focused review of the published literature was performed to contextualize the present case and identify clinically relevant considerations in the diagnosis and surgical management of parapharyngeal schwannomas. Relevant publications were identified through searches of PubMed, Scopus, and Web of Science, with particular attention to clinical presentation, imaging characteristics, histopathological findings, surgical approaches, postoperative complications, and reported recurrence. The reference lists of the retrieved articles were subsequently screened to identify further relevant publications as seen in Table 2 The literature was synthesized narratively rather than as a systematic review or quantitative meta-analysis. Given the predominance of case reports and retrospective case series, the available evidence was considered primarily descriptive and hypothesis-generating. A systematic-review methodology, including PRISMA reporting and a formal risk-of-bias assessment, was not adopted because this manuscript is a case report: the literature review serves to contextualize the clinical and surgical findings of the presented case rather than to answer a pre-specified review question through an exhaustive, reproducible search, and a systematic framework would therefore overstate the methodological formality of this component.

### 3.1. Epidemiology and Nerve of Origin

Across the published literature, PPS tumors consistently represented approximately 0.5% of head and neck neoplasms, roughly half of salivary origin, and about 20% neurogenic, with schwannoma being the most frequent benign nerve-sheath tumor of the space. Large institutional series confirmed this prominence: schwannoma was the single most common parapharyngeal tumor in a 389-case cohort (38.3%) [13] and in a 167-patient cohort (42%) [14], the third commonest benign lesion in a 98-case series [35], the most common neurogenic tumor in a dedicated neurogenic-tumor series [36], and second only to paraganglioma in a lateral-skull-base referral series [37]. In one series it accounted for all 16 post-styloid neurogenic tumors, every schwannoma being post-styloid while salivary tumors were pre-styloid [38]. Schwannoma-only cohorts likewise identified the parapharyngeal space as the most common site of non-vestibular head and neck schwannoma [39,40], accounting for 26.6% of 45 extracranial lesions in one analysis [16].

In the post-styloid compartment the vagus nerve was the single most common parent nerve, followed by the cervical sympathetic chain; within schwannoma-specific parapharyngeal cohorts the vagus (58%) was marginally more frequent than the sympathetic chain (42%) [18], and dedicated series consistently delineated post-styloid subgroups dominated by these two nerves [41]. Given the characteristic displacement of the major vessels, the present case is consistent with these literature reports, although the origin nerve was not directly identified. Pre-styloid neurogenic tumors more often arise from branches of the trigeminal nerve, as illustrated by a mandibular (V3) schwannoma presenting with jaw numbness and taste disturbance [42,43]. Rarer parent nerves were nonetheless well documented, including the glossopharyngeal nerve [44,45]—which predominated (47%) among retro-styloid superior tumors involving the jugular foramen [27]—the accessory nerve [46], the external branch of the superior laryngeal nerve [47] and, recurrently, the hypoglossal nerve [48,49,50]; in a proportion of cases the origin nerve could not be determined [34]. Historical series established the post-styloid predominance early, 12 of 14 parapharyngeal neurilemmomas being post-styloid [15], and had already recognized the vagus, hypoglossal and sympathetic chain as parent nerves within the space, sympathetic lesions producing postoperative Horner syndrome [51,52]; the parapharyngeal space had long accounted for a notable share of head and neck neurilemmomas [53]. Presentation outside the classic 30–70-year-old range was repeatedly reported, in adolescents, young adults [54,55,56] and in children, including sympathetic-chain lesions [57,58,59]. Uncommon variants such as plexiform schwannoma [29] and rare multifocal sympathetic-chain neurinomas confined to the space [60] were also described. Malignant transformation is exceptional, and the great majority of PPS schwannomas are benign and slow-growing.

### 3.2. Clinical Presentation

The characteristic presentation was a slowly enlarging, painless mass of the upper neck and/or a submucosal bulge of the lateral oropharyngeal wall or tonsillar fossa, frequently discovered incidentally or only after reaching a substantial size [55,61]. Because most schwannomas remain clinically silent for years, the duration of symptoms before diagnosis was often long; some lesions were entirely asymptomatic and were resected only because of carotid compression and attendant stroke risk [62], and others were discovered incidentally at a routine dental referral [63] or during intubation [64]. Pressure-related symptoms—dysphagia, globus sensation, snoring or obstructive symptoms and a muffled “hot-potato” voice—were common with larger lesions.

Neurological features were less frequent and depended on the nerve of origin. Vagal lesions could produce hoarseness or vocal-fold paresis, and a cough elicited by palpation was a recognized clue to vagal origin [65,66]; occasional vagal tumors produced atypical autonomic features such as persistent bradycardia and tongue fasciculations [67]. Tongue hemiatrophy signalled a hypoglossal origin [68], whereas high poststyloid masses could produce multiple lower-cranial-nerve palsies, including Collet–Sicard syndrome (cranial nerves IX–XII) [69]. Preoperative Horner syndrome—uncommon before surgery—pointed to sympathetic-chain involvement, including in children [70,71], and first-bite syndrome was likewise recurrently reported in sympathetic-chain tumors [33,72]. Uncommon presentations included pulsatile, hypervascular masses mimicking vascular tumors, described even in children [73,74], and rare acute presentations with inflammation, stridor and airway obstruction [75]. In children the lesion could be mistaken repeatedly for reactive lymphadenopathy or lymphoma [76], and neuroblastoma was the principal differential, excluded by normal urinary catecholamines [58].

### 3.3. Imaging Findings

Contrast-enhanced CT and MRI scans were the cornerstone of preoperative evaluation, defining the compartment of origin, the extent of the tumor and, critically, its relationship to the carotid sheath. Schwannomas typically appeared as well-circumscribed, solitary lesions medial to the carotid sheath, hypo- to iso-attenuating on CT with variable and often modest enhancement, and heterogeneously hyperintense on T2-weighted MRI with inhomogeneous post-gadolinium enhancement. Routine MRI reporting proved highly reliable for parapharyngeal schwannoma, with radiologic–pathologic concordance reaching κ = 0.912 [20], and conventional multiparametric MRI distinguished schwannoma from pleomorphic adenoma with an accuracy of 94.6%, dynamic contrast-enhanced and diffusion-weighted sequences adding little [77]. Intrinsic signal characteristics complemented vessel-based criteria: diffusion-weighted imaging revealed a distinctive “reverse target sign” and characteristic apparent diffusion coefficient (ADC) values separating schwannoma from its mimics [21], and ADC histogram analysis (skewness and kurtosis) separated schwannoma from pleomorphic adenoma even when the mean ADC did not [78].

The principal contribution of imaging is preoperative prediction of the nerve of origin from the pattern of vessel displacement, summarized in Table 3. The seminal rule distinguishes a vagal origin, which separates the ICA from the IJV, from a sympathetic-chain origin, which displaces both vessels together without separating them [18,71,72,79]; a glossopharyngeal tumor displacing the carotid postero-laterally is a recognized exception [45]. The jugular–carotid separation sign distinguished vagal from sympathetic origins with complete accuracy in one schwannoma-specific cohort, outperforming diffusion-weighted MR neurography [80], and quantitative refinement by the carotid–jugular angle stratified vagal (≥100°) from cervical sympathetic (<38.5°) origins [19]. Sympathetic-chain tumors were classically shown to displace the internal and external carotid arteries anteriorly, producing a bird-beak configuration [77,81,82].

Diagnostic pitfalls were nonetheless recurrent. Carotid displacement alone proved an unreliable discriminator, producing a carotid-body-tumor–like lyre sign in a substantial minority [28]; the lyre sign was likewise produced by sympathetic-chain and hypoglossal schwannomas [48,83]. Marked cystic degeneration mimicked cystic metastasis, cystic hygroma or paraganglioma [46,84], ancient schwannomas with cystic, non-enhancing appearances mimicked lymphangioma [85], and hypervascular lesions with internal-carotid splaying were misread as paraganglioma [65,86]. Schwannomas were also confused with carotid body tumors, deep neck abscesses and other cystic lesions [44,47,49,87]. Enhancement pattern was the key discriminating feature: most schwannomas are hypovascular despite splaying the carotid vessels, in contrast to the homogeneously enhancing, artery-encasing paraganglioma [56,88], although occasional hypervascular lesions required preoperative embolization [11,89]. Cross-sectional imaging also corrected clinical and cytological misdiagnoses, disclosing the true post-styloid location of tumors that had masqueraded as parotid lesions [90].

The principal imaging lesson from the present case is that CT and MRI should be regarded as complementary tools for anatomical localization and characterization rather than as definitive methods for identifying the parent nerve. In this case, CT narrowed the differential diagnosis and guided surgical planning but did not reliably distinguish schwannoma from paraganglioma or establish the nerve of origin.

### 3.4. Histopathology and Diagnosis

FNA cytology was frequently non-diagnostic in PPS schwannoma because of the deep location, the paucicellular aspirate and the risk to adjacent neurovascular structures; the definitive diagnosis was therefore almost always established histopathologically after excision. Diagnostic yield varied markedly across series—from non-diagnostic in individual reports [89] to a correct preoperative diagnosis in every case of one institutional cohort [36]—and was around 30% in vagal schwannomas and in neurogenic aspirates generally [38,91]. Cytology was sometimes contributory by revealing spindle cells [92] but frequently misleading, being read as pleomorphic adenoma [79], deliberately avoided in hyperivascular lesions [74], or inconclusive to the point of mandating incisional biopsy [63]; image-guided core-needle biopsy provided histological confirmation in selected tumors [93]. Early experience delivered a durable cautionary lesson: blind incisional or transoral biopsy of a parapharyngeal mass could precipitate torrential hemorrhage or internal-carotid injury, arguing for an imaging-led diagnostic pathway rather than blind sampling [59,94].

Histologically, schwannomas are typically composed of alternating cellular Antoni A areas, with nuclear palisading and Verocay bodies, and hypocellular Antoni B areas [95]. S-100 and SOX10 are commonly expressed in Schwann-cell tumors [54] and can support the diagnosis when interpreted together with the morphological findings and, when necessary, additional immunohistochemical markers. In the present case, the tumor showed diffuse strong S-100 positivity, while SOX10 immunohistochemistry was not performed. The low Ki-67 proliferation index supported the benign histopathological interpretation but was not considered independently diagnostic [31,61]. These findings, together with the characteristic morphology, supported the final diagnosis of benign schwannoma [94,96].

### 3.5. Management and Surgical Approach

Complete surgical excision remains the principal treatment for symptomatic or enlarging PPS schwannomas and is generally associated with favorable local tumor control. However, the available evidence is based predominantly on retrospective series and case reports, with variable duration and completeness of follow-up. The transcervical approach was the most frequently used and was preferred for post-styloid lesions; transoral routes, including endoscopically assisted and transoral-robotic techniques, were used for selected pre-styloid or well-encapsulated tumors with favorable vessel displacement; and trans-parotid, trans-mandibular, trans-zygomatic or combined approaches were reserved for large tumors or those extending toward the skull base. Observation and stereotactic radiosurgery were reported as alternatives in selected patients who were poor surgical candidates or who declined surgery, with CyberKnife radiosurgery offering a non-surgical option for deep, high-risk lesions [67]. A structured diagnostic and therapeutic algorithm has been proposed for vagal schwannoma [26]. No single radiological criterion defines inoperability in PPS schwannoma; respectability instead depends on tumor biology, anatomical relationships, vascular involvement, and the anticipated morbidity of treatment. A lesion may be considered technically unresectable when safe complete removal cannot be achieved without unacceptable morbidity, particularly with extensive vascular encasement, unreconstructable involvement of critical neurovascular structures, or skull-base extension that cannot be safely accessed; in such circumstances, individualized multidisciplinary assessment and, where appropriate, observation or radiosurgery may be considered [67]. In the present case, given the large tumor size, a modified Blair incision extended cervically and submandibularly was performed to obtain better access to the post-styloid area.

Several recent observational series have explored nerve-sparing intracapsular approaches, particularly for tumors in which the parent-nerve fascicles are closely related to the capsule [66]. Some studies report fewer non-nerve complications and lower rates of first-bite syndrome in case of intracapsular enucleation compared with more extensive resection [24,91,97,98]. However, these comparisons are subject to selection bias and confounding by indication, as tumors selected for intracapsular or minimally invasive approaches may differ anatomically from those requiring radical excision. Furthermore, follow-up and recurrence reporting are inconsistent, and long-term equivalence in disease control has not been established. The plexiform variant is an explicit exception, recurring after nerve-sparing resection and warranting complete excision [29].

Endoscopic and robotic techniques have been increasingly reported in selected patients, with the aim of limiting tissue trauma and neurovascular morbidity [13]. The available comparative evidence is observational, however, and does not establish that the surgical route itself reduces complications. In the largest schwannoma-specific comparative series (85 retro-styloid tumors), endoscope-assisted transoral resection achieved the shortest operative time, the least drainage and the lowest complication rate, with no posterior cranial-nerve injury [23]; endoscopic-assisted transoral approaches were likewise validated for benign, encapsulated, non-vascular tumors showing posterior vessel displacement [22,99]. Other reported possibilities include a combined transcervical–transoral route for large lesions [100], transoral video-laryngoscopic removal of a giant high-cervical tumor without mandibulotomy [93], endoscopic transoral removal of retro-styloid tumors with pre-emptive internal-carotid control [101], robotic-assisted retro-auricular resection [86], transoral robotic surgery via a “J”-shaped incision for retro-styloid vagal and sympathetic tumors [102], and scar-free, capsule-preserving transoral and robotic resections without first-bite syndrome for selected pre-styloid tumors [103,104,105]. An endoscopic endonasal approach to a sympathetic-chain schwannoma spared the chain and avoided Horner syndrome [106].

Mandibulotomy-sparing strategies matured in parallel. Intracapsular ultrasonic-aspirator debulking [107] and microdebrider cavitation [108] reduced tumor volume sufficiently to permit purely transcervical removal of high sympathetic tumors, albeit with Horner and first-bite syndrome in all four patients in the latter series; a mandibulotomy-sparing transcervical approach in 44 tumors yielded only two permanent cranial-nerve palsies among ten schwannomas [109]; a minimal transcervical approach spared the submandibular gland, parotid and mandible with a mean one-day stay and no recurrence [92]; and extracapsular dissection through a short incision suited selected small tumors distant from the skull base [110]. Conversely, giant tumors abutting the ICA [43,111], skull-base-reaching lesions [32,37,112,113] and mucosa-violated tumors requiring narrow-field transoral oropharyngectomy [114] still warranted osteotomy or lateral-skull-base corridors, sometimes with preoperative embolization or internal-carotid stenting [37], and a mandibular-swing osteotomy remained an option for complex lesions [115]. An extracranial anterolateral approach achieved gross-total resection in 89.4% of jugular-foramen-involving schwannomas without craniotomy [27]. Adjunctive refinements included intraoperative nerve-integrity monitoring to localize the parent nerve, with greater-auricular-nerve grafting when the nerve was unsalvageable [116]; immediate grafting with electromyographically confirmed reinnervation when a nerve segment had to be sacrificed [53]; intracapsular coblation debulking under a structured classification [105]; and pedicled submandibular-gland-flap reconstruction of the resulting parapharyngeal dead space [117]. The foundational principle set out in the earliest series—external transcervical excision, with an explicit warning against transoral removal—remains the reference point against which these newer corridors are judged [58,118].

### 3.6. Outcomes and Complications

Reported local tumor control was generally excellent in the available literature, with a very low recurrence risk; however, follow-up duration and completeness varied considerably, and recurrence was not consistently assessed or reported. Complete excision yielded no recurrence over follow-up of up to 108 months [72] and of two years or more in other cohorts [41,92]; a large transcervical series achieved 95% complete excision with 1% recurrence [14], the largest comparative series reported an overall recurrence rate of 2.4% [23], and individual reports confirmed complete removal without new neurological deficit [60,119]. Malignant peripheral nerve sheath tumor arising in association with a conventional benign schwannoma is exceedingly uncommon. Reports of malignant peripheral nerve-sheath tumors involving the parapharyngeal region should therefore not be interpreted as evidence of a routine progression pathway from conventional schwannoma [120]. Length of hospital stay was typically a few days.

Functional outcomes, by contrast, were dominated by the parent-nerve deficit. Findings varied appreciably between comparative series: a smaller comparative series of 21 patients reported parent-nerve deficit in all patients regardless of extirpation technique [5], whereas the largest retro-styloid comparative series (n = 85) reported no posterior cranial-nerve injury with an endoscope-assisted transoral approach [23], indicating that outcomes are technique- and cohort-dependent rather than uniformly poor. Deficits were nerve-specific. Vagal resection frequently incurred dysphonia and dysphagia, generally resolving with rehabilitation after nerve-preserving excision [95,121,122] but persisting after total resection: recurrent laryngeal palsy occurred in 4 of 10 and hoarseness in 6 of 10 patients in one vagal series [26], permanent vocal-fold paralysis followed total excision despite medialization thyroplasty [65,107], and combined vocal-fold and hypoglossal deficits [123] or palsy requiring primary neurorrhaphy [124] were also reported. Horner syndrome was the essentially unavoidable consequence of sympathetic-chain sacrifice, in adults and children alike [15,72,81,82,89,108,125], usually permanent [71,96,126] though occasionally transient [74] and generally well tolerated, improving symptomatically over months [127]. In selected sympathetic-chain or parapharyngeal tumors involving the relevant sympathetic pathways, the surgical extent and preservation or disruption of associated structures may influence the risk of first-bite syndrome; however, this relationship should not be generalized to all PPS schwannomas [128,129]. In cervical sympathetic-chain tumors overall, Horner syndrome (57%) and first-bite syndrome (33%) predominated [28].

Lower-cranial deficits could occur even when nerve preservation was attempted [73], and vagal enucleation more often preserved cord function with only occasional transient palsy [66]. Rare but serious vascular and autonomic complications were also reported, including perioperative stroke with contralateral hemiplegia after en bloc excision of a poststyloid tumor [30] and severe intraoperative bradycardia and hypotension provoked by manipulation of a vagal tumor, reversed with atropine [64]. These nerve-specific patterns—vocal-cord paralysis with pharyngolaryngeal anesthesia for vagal tumors, Horner syndrome for sympathetic ones—had already been catalogued in the earliest neurogenic-tumor series and have remained consistent across five decades [130,131].

## 4. Discussion

Taken together, the published literature on parapharyngeal schwannoma confirms several consistent themes. First, although PPS tumors are rare and histologically heterogeneous, the schwannoma is a leading benign neurogenic lesion of the space, most often arising from the vagus nerve or the cervical sympathetic chain in the post-styloid compartment. Second, the clinical presentation is typically indolent and non-specific, so that a high index of suspicion and appropriate cross-sectional imaging are essential. Third, imaging may provide useful clues regarding the compartment of origin and the relationship of a parapharyngeal tumor to the carotid and jugular vessels; however, these findings do not reliably establish the parent nerve in every case. In the present case, despite detailed contrast-enhanced CT evaluation, the nerve of origin could not be determined.

Contemporary surgical management increasingly emphasizes preservation of functional nerve fascicles when anatomically feasible. Observational studies suggest that nerve-sparing intracapsular approaches may reduce selected postoperative functional complications in appropriately selected tumors. However, comparisons with more extensive resection are subject to selection bias and confounding by indication, and the available literature does not establish long-term equivalence in disease control. A parent-nerve deficit remains common even with meticulous technique, because the functioning fascicles are frequently splayed over the tumor capsule. This must be communicated explicitly during informed consent, and intraoperative nerve monitoring may assist in identifying and preserving functional fascicles. The choice of approach is dictated by compartment, size and vascular relationships: the transcervical route remains the workhorse for post-styloid lesions, offering proximal and distal vascular control, whereas endoscopically assisted transoral and transoral-robotic techniques are increasingly used for selected, well-encapsulated tumors that displace the great vessels posteriorly, avoiding external incisions and shortening hospital stay. Larger tumors and those approaching the skull base may still require trans-parotid, trans-mandibular or combined approaches.

Diagnostic confirmation continues to rely on histopathology, since FNA is often non-diagnostic in this deep location. The combination of Antoni A and B architecture with Verocay bodies and diffuse S-100 positivity supports the diagnosis and, interpreted together with morphology, helps distinguish schwannoma from other PPS lesions such as pleomorphic adenoma, paraganglioma and malignant nerve-sheath tumors [53].

The present case exemplifies several of these themes. The tumor followed the characteristic indolent course, having been observed for 7–8 years before slow enlargement and pain prompted intervention, and presented as a well-defined mass of the submandibular and parotid region rather than with cranial-nerve deficits.

The preoperative imaging assessment was based on contrast-enhanced CT scan, which is routinely used as the initial cross-sectional imaging modality for parapharyngeal lesions in our clinical practice. The examination demonstrated a well-circumscribed and clearly delineated post-styloid mass without evidence of local invasion or other aggressive radiological features. Although the initial radiological impression included paraganglioma in the differential diagnosis, the lesion did not demonstrate features that, in the clinical context, were considered sufficiently suggestive of a highly vascular tumor to warrant additional vascular imaging. Given its well-defined, apparently non-invasive appearance, MRI was not considered necessary for further immediate diagnostic or surgical planning.

Two common diagnostic pitfalls were encountered. Lateral extension toward the parotid raised the possibility of a parotid neoplasm, however cross-sectional imaging ultimately disclosed a true post-styloid origin [90]—and the post-styloid location with heterogeneous enhancement and marked carotid–jugular displacement prompted a preoperative suspicion of paraganglioma, reflecting the enhancement-based pitfall in which lyre-type carotid splaying is shared but the hypovascular enhancement of schwannoma is the distinguishing feature [46,56,83,84]. The marked vascular displacement on preoperative CT was nonetheless the key to anticipating a neurogenic origin [72,77]; it is the qualitative counterpart of the jugular–carotid separation sign [80] and of the vessel-displacement rules [18] that quantitative tools such as the carotid–jugular angle now seek to formalize [19]. FNA was not performed in the present case, therefore, the discussion of the limited diagnostic yield of FNA refers exclusively to findings reported in the literature and not to the present patient’s diagnostic work-up [38,63], however the literature recommends caution against blind biopsy of parapharyngeal masses [94] reinforcing the imaging-led pathway we followed.

Intracapsular enucleation performed without capsular damage, as described in the cited literature, has been associated with favorable local tumor control, although the duration and completeness of follow-up varied considerably across studies. In the present case, complete removal of the intact encapsulated tumor was achieved without capsular rupture. Although this resulted in favorable clinical follow-up, the procedure should not be interpreted as formal intracapsular nerve-sparing enucleation because the parent nerve was not identified. Observational studies suggest that nerve-sparing intracapsular techniques may be associated with improved functional outcomes and fewer non-nerve complications in selected patients; however, the available evidence is heterogeneous and does not establish long-term equivalence in disease control with more extensive resection [5,23,24,30,91,95,98,128]. Comparisons between intracapsular and more extensive resection are also subject to confounding by indication, because tumors selected for more extensive surgery may have greater size, anatomical complexity, vascular involvement, or functional-nerve involvement. The case also illustrates two limitations frequently encountered in real-world practice. First, the parent nerve could not be identified intraoperatively. In retrospect, this is precisely the situation in which intraoperative nerve-integrity monitoring is of greatest value, since a stimulus probe allows functional fascicles tethered to the capsule to be mapped and a safe plane of dissection to be selected before the capsule is opened [115]; where the parent nerve must nevertheless be sacrificed, immediate interposition grafting can restore continuity [55]. Systematically incorporating nerve monitoring into the operative protocol for poststyloid parapharyngeal masses would allow the nerve of origin to be documented prospectively rather than inferred, and is the principal change we would make to our own practice. Second, follow-up was clinical only, so recurrence and vocal-fold mobility were assessed symptomatically rather than by imaging and endoscopy; adding a baseline postoperative laryngoscopy and a single cross-sectional study at one year would allow subclinical deficits and residual disease to be excluded objectively. No clinically apparent neurological deficit was identified at 6- and 12-month follow-up. Because the parent nerve was not identified intraoperatively and postoperative flexible laryngoscopy was not performed, anatomical or subclinical functional preservation cannot be established.

A recent systematic review by Chiari et al. specifically addressed the surgical management of parapharyngeal vagal schwannoma and proposed practical considerations for approach selection [25]. The present review differs from and complements that work in two respects: rather than being restricted to vagal tumors in surgically treated patients, it encompasses parapharyngeal schwannoma irrespective of the nerve of origin—including the vagus nerve, the cervical sympathetic chain, the accessory nerve and cases in which the parent nerve could not be determined—and it integrates an original, illustrated case report. This broader scope provides a broader narrative overview across different nerves of origin of the clinical, imaging, histopathological and surgical spectrum of the entity while remaining consistent with the approach-selection principles emphasized in vagal-specific analyses.

### Limitations

Several limitations must be acknowledged. The literature review also has several methodological limitations. Because the focused search was initially conducted by a single reviewer, relevant publications may have been missed despite subsequent review of reference lists. Potential overlap between publications from the same institutions could not always be excluded from the published information and may result in repeated reporting of individual patients. In addition, some observations concerning surgical technique and functional outcomes derive from cervical or mixed head-and-neck schwannoma populations rather than PPS-specific cohorts and should therefore be interpreted as contextual rather than directly generalizable evidence. Neurological outcomes were assessed inconsistently across reports, and recurrence was frequently not reported or was reported after variable follow-up intervals. Consequently, the apparent low recurrence rate should not be interpreted as a reliable population-level estimate. The literature review was not prospectively registered and no protocol was publicly deposited. The published evidence on parapharyngeal schwannoma is dominated by retrospective case series and individual case reports, which carry an inherent risk of selection and reporting bias. Quantitative pooling was not attempted. These designs do not intrinsically preclude synthesis; rather, marked clinical and methodological heterogeneity, possible overlapping cohorts, selective reporting, inconsistent outcome definitions and incomplete follow-up would render any pooled estimate unreliable in this dataset. The restriction of the literature search to English-language, indexed publications may introduce language and publication bias.

Finally, the accompanying case is a single observation: its 12-month follow-up was clinical and cannot exclude subclinical neurological or radiological findings, and the lessons drawn from it are illustrative rather than generalizable.

## 5. Conclusions

Parapharyngeal schwannomas are rare benign tumors that may reach considerable size before becoming clinically apparent. Their deep location and close relationship with major vascular and neural structures can make both diagnosis and surgical management challenging. In post-styloid lesions, imaging may demonstrate characteristic vascular displacement and anatomical relationships but may not reliably establish the nerve of origin or distinguish schwannoma from paraganglioma in every case.

In the present case, contrast-enhanced CT scan demonstrated a large, well-circumscribed and apparently non-invasive post-styloid mass that was considered radiologically compatible with paraganglioma. The lesion was subsequently diagnosed as a benign schwannoma following complete surgical excision. The origin nerve was not identified intraoperatively, and the absence of a clinically apparent postoperative neurological deficit should therefore not be interpreted as definitive evidence of anatomical nerve preservation.

The focused literature review indicates that surgical excision remains the principal treatment for symptomatic or enlarging PPS schwannomas, with generally favorable local control reported in the available literature. However, the evidence is largely based on case reports and retrospective series, with substantial heterogeneity in surgical techniques, neurological outcome assessment, and follow-up duration. Nerve-sparing intracapsular approaches may provide functional advantages in selected patients, but current evidence is insufficient to establish their long-term equivalence in disease control with more extensive resection. Individualized assessment of tumor anatomy, vascular relationships, anticipated nerve involvement, and the patient’s functional priorities therefore remains central to surgical planning.

## Figures and Tables

**Table 1:** Key radiological differentiators of the pre- and post-styloid space [2,3].

Radiographic Sign	Prestyloid Mass	Retrostyloid (Poststyloid) Mass
Internal Carotid Artery (ICA)	Displaced posteriorly or postero-laterally	Displaced anteriorly or antero-medially
Parapharyngeal Fat Pad	Displaced posteriorly or postero-medially	Displaced anterolaterally
Styloid Process	Displaced posteriorly	Displaced anteriorly

**Table 2:** Selected published reports of parapharyngeal schwannomas reviewed for contextualization of the present case.

Study (Journal, Year)	Design/n	Key Findings
Sato et al., Head Neck 2018 [5]	Retrospective case series; n = 21 PPS schwannomas	Parent-nerve deficit in all patients regardless of technique; first-bite syndrome less frequent after intracapsular enucleation than total resection (40% vs. 100%; *p* = 0.045); non-nerve complications 0% vs. 42.9%.
Jiang et al., Curr. Oncol. 2023 [2]	Single-center series and literature review	Schwannoma and pleomorphic adenoma are the most common PPS lesions; transcervical approach in the majority; no disease-related mortality reported during follow-up.
Liu et al., Front. Surg. 2026 [8]	Retrospective case series; n = 15 (1998–2023)	Painless neck mass is the most common presentation (53%); CT/MRI vessel-displacement patterns were used to infer the probable nerve of origin, without independent intraoperative confirmation in all cases; Horner syndrome in 1 of 9 operated patients; no recurrence.
Zhu et al., Eur J Surg Oncol 2023 [13]	Surgical-trends series; n = 389 PPS tumors	Schwannoma is the single most common PPS tumor (38.3%); increasing reported use of minimally invasive endoscopic and robotic approaches over the study period.
Shi et al., Acta Otolaryngol 2016 [14]	Retrospective 10-year series; n = 167 PPS tumors	Schwannoma is the most common (42%); transcervical approach in 84%, 95% complete resection, 1% recurrence; intraoperative endoscopy for residual tumor.
Hamza et al., Arch Otolaryngol Head Neck Surg 1997 [15]	Dedicated PPS case series; n = 14	Twelve of 14 post-styloid; all sympathetic-chain tumors developed postoperative Horner syndrome.
Zorlu et al., 2025 [16]	Retrospective series; n = 45 extracranial head and neck schwannomas	Parapharyngeal region is the single most common site (26.6%).
Anil and Tan, AJNR Am J Neuroradiol 2010 [17]	Imaging series; n = 12 sympathetic-chain schwannomas	Sympathetic-chain tumors displace ICA and IJV together without splaying; T2-bright, heterogeneous enhancement, medial to the carotid sheath.
Saito et al., Arch Otolaryngol Head Neck Surg 2007 [18]	Imaging series; n = 12 PPS schwannomas	Vagal tumors separate the ICA from the IJV (ICA anteromedial); sympathetic-chain lesions displace both vessels together—the key preoperative discriminator.
Wong et al., Int. J. Surg. 2023 [19]	Single-center cohort (n = 17) plus pooled literature (n = 88)	Quantitative predictor of the parent nerve: vagus if the angle is ≥100°, cervical sympathetic chain if <38.5°.
Alshahrani et al., J Clin Med 2026 [20]	Diagnostic-accuracy study; n = 8 (schwannoma subgroup)	Routine MRI reporting highly reliable for parapharyngeal schwannoma (κ = 0.912).
Das et al., Indian J Radiol Imaging 2016 [21]	Imaging series; n = 12 (8 parapharyngeal)	Distinctive “reverse target sign” and characteristic ADC values distinguish schwannoma from its mimics on diffusion-weighted MRI.
Orlando et al., 2023 [22]	Case series; n = 7 (3 transcervical, 4 endoscopy-assisted transoral)	Transcervical route for post-styloid and transoral for pre-styloid lesions; the transoral route is valid for benign, encapsulated, non-vascular tumors with posterior vessel displacement; no recurrence.
Zhang et al., Head Neck 2024 [23]	Comparative series; n = 85 retro-styloid schwannomas	Endoscope-assisted transoral route: shortest operative time, least drainage, lowest complication rate, no posterior cranial-nerve injury; overall recurrence 2.4%.
Kampel et al., J Otolaryngol Head Neck Surg 2023 [24]	Retrospective cohort; n = 25 (13 parapharyngeal)	Intracapsular (nerve-sparing) resection preserved function in 61% vs. 0% after en bloc removal.
Chiari et al., J Laryngol Otol 2026 [25]	Systematic review and meta-analysis	Function-sparing subtotal or intracapsular resection preserves nerve function and lowers the rate of vocal-fold palsy versus radical resection with anastomosis.
Loperfido et al., Medicina 2023 [26]	Case series (n = 10) and literature review	Supports imaging-guided diagnosis and a nerve-preserving surgical strategy; recurrence rare after complete or near-complete excision.
Gallo et al., World Neurosurg 2019 [27]	Retrospective series; n = 38 retro-styloid superior PPS	Extracranial anterolateral approach for jugular-foramen-involving tumors: gross-total resection in 89.4%, no craniotomy, low morbidity.
Langerman et al., Head Neck 2013 [28]	Retrospective series; n = 24 (10 schwannomas)	Horner syndrome 57%, first-bite syndrome 33%; carotid displacement an unreliable discriminator (lyre sign in 38%).
Ijichi et al., Auris Nasus Larynx 2018 [29]	Case report; n = 1	Recurrent plexiform schwannoma recurs after nerve-sparing resection and warrants complete excision—a subtype-specific exception to enucleation.
Shaikh et al., Eur Arch Otorhinolaryngol 2023 [30]	Case report; n = 1	Perioperative stroke with contralateral hemiplegia after en bloc excision—a rare but dreaded complication.
Budu et al., Rom J Morphol Embryol 2015 [31]	Case report; n = 1	S-100/SOX10 positivity with low Ki-67 supported a benign interpretation despite degenerative atypia.
Ferreira et al., 2020 [32]	Case report; n = 1	Large parapharyngeal schwannoma with non-diagnostic FNA, confirmed by S-100/SOX10-positive histology with Antoni A/B areas and Verocay bodies.
Senjab et al., 2025 [33]	Case report; n = 1	Cervical sympathetic-chain schwannoma in the parapharyngeal space, with imaging and histopathological correlation.
Abd El-Fattah et al., 2026 [34]	Case series; multiple anatomical sites	Case series of extracranial non-vestibular head and neck schwannomas illustrating diverse anatomical locations and surgical approaches, including parapharyngeal sites.

**Table 3:** Imaging-based prediction of the nerve of origin of post-styloid parapharyngeal schwannomas from vessel-displacement patterns.

Feature	Vagal Schwannoma	Cervical Sympathetic-Chain Schwannoma
Relationship of ICA and IJV	Splayed and separated—the tumor lies between the vessels	Displaced together, without separation
Typical ICA displacement	Anteromedial displacement of the ICA	Lateral displacement of ICA and IJV as a unit
Compartment	Post-styloid PPS	Post-styloid PPS (more medial and posterior)
Associated clinical sign	Vocal-fold paresis or hoarseness if the nerve is involved	Horner syndrome (ptosis, miosis, anhidrosis)

ICA, internal carotid artery; IJV, internal jugular vein; PPS, parapharyngeal space.

## Data Availability

All data supporting the findings of this case report are contained within the article. No separate study-level dataset was generated or analyzed, and no additional data are held by the authors.

## Abbreviations

The following abbreviations are used in this manuscript:

ADC	apparent diffusion coefficient
CI	confidence interval
COPD	chronic obstructive pulmonary disease
CT	computed tomography
FNA	fine-needle aspiration
ICA	internal carotid artery
IJV	internal jugular vein
MRI	magnetic resonance imaging
PPS	parapharyngeal space
